# Electrical Permittivity and Conductivity of a Graphene Nanoplatelet Contact in the Microwave Range

**DOI:** 10.3390/ma11122519

**Published:** 2018-12-11

**Authors:** Stefano Bellucci, Antonio Maffucci, Sergey Maksimenko, Federico Micciulla, Marco D. Migliore, Alesia Paddubskaya, Daniele Pinchera, Fulvio Schettino

**Affiliations:** 1 INFN-Laboratori Nazionali di Frascati, Via E. Fermi 40, 00044 Frascati, Italy; maffucci@unicas.it (A.M.); federico.micciulla@lnf.infn.it (F.M.); 2 Department of Electrical and Information Engineering, University of Cassino and Southern Lazio, via G. Di Biasio 43, 03043 Cassino, Italy; mdmiglio@unicas.it (M.D.M.); pinchera@unicas.it (D.P.); schettino@unicas.it (F.S.); 3 Institute for Nuclear Problems, Belarusian State University, Minsk, Belarus, Bobruiskaya str. 11, 220030 Minsk, Belarus; sergey.maksimenko@gmail.com (S.M.); paddubskaya@gmail.com (A.P.); 4 Tomsk State University, 36 Lenin Ave., 634050 Tomsk, Russia

**Keywords:** graphene nanoplatelets, nanocomposite, permittivity, scattering parameters

## Abstract

This paper investigates the electrical properties in the microwave range of a contact made by graphene nanoplatelets. The final goal is that of estimating the range of values for the equivalent electrical complex permittivity of a contact obtained by integrating low-cost graphene in the form of nanoplatelets (GNPs) into a high-frequency electrical circuit. To this end, a microstrip-like circuit is designed and realized, where the graphene nanoplatelets are self-assembled into a gap between two copper electrodes. An experimental characterization is carried out, both to study the structural properties of the nanomaterials and of the realized devices, and to measure the electromagnetic scattering parameters in the microwave range by means of a microstrip technique. A full-wave electromagnetic model is also derived and used to investigate the relationship between the measured quantities and the physical and geometrical parameters. The combined use of the experimental and simulation results allows for retrieving the values of the equivalent complex permittivity. The equivalent electrical conductivity values are found to be well below the values expected for isolated graphene nanoplatelets. The real part of the electrical relative permittivity attains values comparable to those obtained with GNP nanocomposites.

## 1. Introduction

In recent decades, the use of nanostructured materials in electronics has been widely investigated, given their exciting features and outstanding properties, potentially suitable to solve many of the open problems for the next generations of electronics technologies [1]. Therefore, an increasing amount of interest has been given to innovative materials and design solutions. 

Among the nanomaterials synthesized and studied so far, a special role is played by those derived from the carbon, such as the graphene or the carbon nanotubes, because of their fascinating behavior and excellent mechanical, thermal and electrical properties [2,3]. The use of carbon-based nanomaterials in electronics has been investigated for decades [4,5,6,7,8], and the proposed applications may be divided in two main categories: (i) nano-carbon as reinforcing material to realize novel composites (nanocomposites); (ii) nano-carbon as alternative material to replace conductors or dielectrics in electronic devices. 

The carbon-based nanocomposites have been shown to provide excellent electrical and electromagnetic performance [9,10,11,12], so that their use to fabricate films, shields or coatings moved from the stage of the lab to the market, assessing a new technology.

On the other hand, the use of carbon materials to replace conductors or dielectrics in electronic devices has not reached the same technology maturity. Indeed, carbon nanotubes (CNTs) and graphene nanoribbons (GNRs) have been proposed, for instance, to replace conventional conductors in nanoscale applications such as on-chip nano-interconnects [13,14], quantum circuits nano-interconnects [15], nano-packages [16,17], nano-transistors [18,19], nano-antennas and nano-sensors [20,21,22]. The main reasons why this technology has not yet reached the commercial stage are related to the cost of the fabrication of nanomaterials with the required quality and of their integration. As for the integration, presently the main limit is due to the growth conditions (e.g., the temperature) that are still not compatible with a monolithic integration of carbon conductors and conventional CMOS technology. Therefore, the integration can be only made by means of cumbersome and expensive transfer techniques.

**Figure 1:**
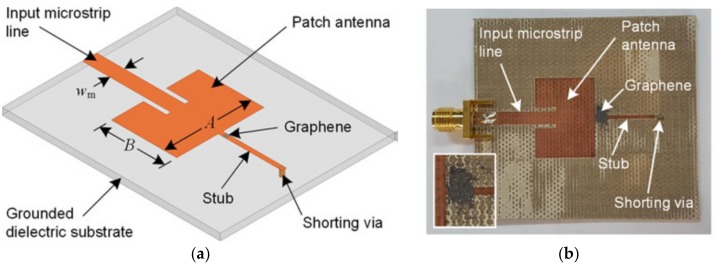
Graphene flakes integrated into a patch antenna [27]: (**a**) geometry; (**b**) picture.

For the above reasons, recently an increasing interest has been paid to some low-cost versions of the graphene, such as the so-called Graphene Nanoplatelets (GNPs), irregular flakes of graphene. Such platelets are typically made by few layers of graphene, hence with a thickness of few nanometers and the other two dimensions of the order of the microns. A GNP can be fabricated with a simpler and cheaper procedure compared to a graphene sheet: for instance, in [23] it is obtained from commercial expandable graphite by means of a thermal expansion and a further sonication process. In addition, the GNPs may be integrated into an electrical circuit by means of a bottom-up self-assembly technique, such as that proposed in [24]. The electrical properties of this low-cost material are of course poor compared to the isolated pure graphene sheet or ribbon, but they can still be suitable for the applications. Indeed, the use of GNPs as fillers in nanocomposites has been proven to be effective in providing good performance in terms of electromagnetic shielding effectiveness, for instance [25]. In addition, the use of GNPs integrated into high-frequency circuits has been successfully proposed for realizing attenuators [26] or tunable antennas, Figure 1 [27].

In view of integrating the GNPs into a high-frequency circuit, it is essential to estimate the achievable values of the equivalent electrical parameters (dielectric constant and electrical conductivity), taking into account not only the nanomaterial features but also the effect of the contact resistance at the interfaces with the conventional metals, which play a major role in determining the final performance of carbon-based nano-circuits [28,29]. Indeed, the measured values of these parameters available in literature refer to isolated GNPs or to GNP nanocomposites [30]. 

The novelty of this paper is the proposal of a technique based on electromagnetic modeling and experimental characterization, able to retrieve the high-frequency equivalent complex permittivity of a GNP contact embedded in copper electrodes. Specifically, such an equivalent parameter accounts for the effects of the GNP/Cu contacts, which are essential in view of correctly analyzing or designing electrical circuits and devices with graphene embedded.

The details of the fabrication and of the experimental characterization of the circuit are given in Section 2, along with the electromagnetic model used to simulate its behavior. The model is based on physical parameters such as the equivalent complex permittivity and the dimensions of the GNP contact.

Section 3 reports the results of: (i) a structural analysis on the circuits, performed by means of Scanning Electron Microscopy; (ii) a sensitivity analysis performed by means of the electromagnetic model; and (iii) an electromagnetic characterization in the microwave range with a Vector Network Analyzer, by means of a microstrip technique. Indeed, this technique is suitable for our purposes, being able to include the contacts, which is not the case for other measurement methods commonly adopted to estimate the permittivity of a material (see for instance [31]). The joint use of the microstrip measurement technique and of the numerical simulation of the full-wave model provide, respectively, the measured and simulated scattering parameters (S-parameters). The equivalent permittivity is finally derived by identifying the parameters of the proposed model, by using the results of the structural characterization and the measured S-parameters. Examples of permittivity measurement methods based on the use of full-wave simulations are provided, for instance, in [32].

The final Section of Conclusions resumes the main results obtained in this paper and outlines some perspective work suggested by the results achieved so far.

## 2. Materials and Methods

### 2.1. Preparation and Structural Characterization of the Sample Circuits

**Figure 2:**
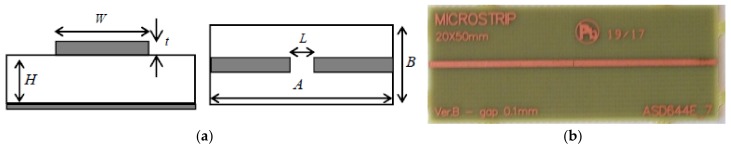
The microstrip test-vehicle: (**a**) geometry; (**b**) picture.

The test-vehicle is the microstrip circuit depicted in Figure 2, in which the copper signal line is cut to realize a gap. The parameters values for the geometry are reported in Table 1. The dielectric material is FR4 (PCL370HR), with relative permittivity between 4.17 and 3.92 in the range (1–10) GHz.

The considered nanomaterial is a powder of commercial graphene nanoplatelets (GNPs), that has been characterized through analytical scanning electron microscopy, performed by means of the SEM microscope LEO/ZEISS, model 1455 VP, Austin, TX, USA), also featuring energy dispersive X-ray spectrometry (EDX). To this end, the powder has been placed on an aluminum stop with double-sided carbon tape. The voltage for the energy dispersive analysis was 20 keV.

These GNPs have been transferred into the microstrip gap, to form a contact between the two copper electrodes. To this end, it has been used a self-assembly technique, recently proposed by the Authors [28]: the GNPs have first been dispersed into isopropyl alcohol, then dropped down in the gap, using a small needle syringe. During this phase, an electric field has been imposed in the gap, by applying a DC voltage source to the ends of the two copper electrodes. Under the action of such a field, the GNPs can be attracted inside the gap, creating the contact after the alcohol evaporation. At this stage, the contact is only due to Van der Waals forces. In order to mechanically fix it, an acrylic spray is used to deposit a film over the GNPs and the copper electrodes. 

The correct integration of GNPs into the gap between the copper lines has been checked by SEM analysis, performed by SEM TESCAN, model VEGA II, Brno–Kohoutovice, Czech Republic.

Two different samples of the above circuit have been fabricated.

### 2.2. Electromagnetic Model

**Figure 3:**
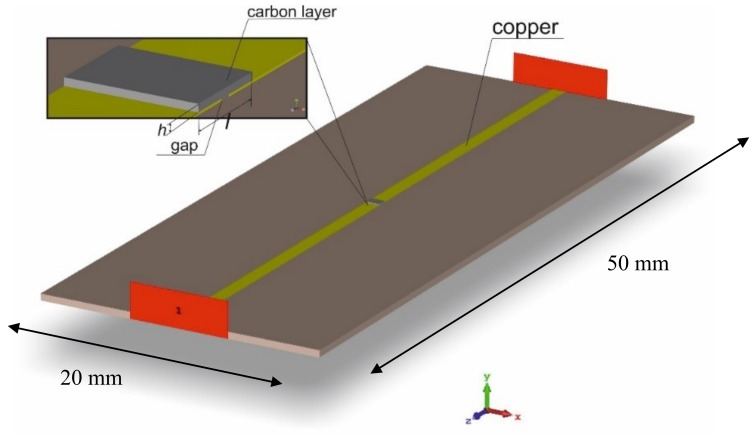
Microstrip geometrical model implemented in CST Microwave Studio, with the waveports highlighted in red. The inset describes the gap with the extra material box.

As outlined above, an electromagnetic model of the circuit is needed to investigate the functional relationship between the measured quantities and the equivalent parameters, taking into account all the phenomena involved in the reflection/transmission process. To this purpose, the microstrip with GNPs has been modeled by means of the simulation tool CST Microwave Studio [33]. The geometry has been designed as in Figure 3. The electrodes and the return ground of the microstrip have been modeled as copper with conductivity of $\sigma_{Cu}=5.8\cdot 10^{7}\;S/m.$ In the considered frequency range, the values of the dielectric constant of the FR4 dielectric has been assigned by linearly interpolating the values $\varepsilon_{r,FR4}=4.17$ at 1 GHz and $\varepsilon_{r,FR4}=3.92$ at 10 GHz. The GNP gap has been modeled by a complex relative permittivity $\varepsilon =\varepsilon^{\prime }+i\varepsilon^{\prime \prime },$ assuming a simple Drude model [34]:

(1) $$\varepsilon =\varepsilon \prime -i\frac{\sigma }{\omega \varepsilon_{0}},$$

that involves a frequency-independent dielectric constant $\varepsilon^{\prime }$ and electrical conductivity σ. 

A frequency-domain simulation is performed by introducing the input and output waveports of the microstrip as shown in Figure 3, in order to compute the scattering parameters, to be compared to the results of the experimental characterization. 

As shown in the next Section, the low-cost self-assembly technique described above cannot provide a strict control on the contact shape, therefore extra material may be found outside the gap volume. Its presence influences the results in the microwave range, therefore it must be considered. Here it is simply described as an extra box of length *l* and height *h* that covers the original gap (inset of Figure 3).

Summarizing, the electromagnetic model is characterized by four parameters that act as degrees of freedom: the two electrical parameters $\varepsilon^{\prime }$ and σ appearing in Equation (1), and the two geometrical parameters *l* and *h* referring to the extra-material box in Figure 3 (inset).

### 2.3. Electromagnetic Characterization

The test-vehicle (the Device Under Test, DUT) described in paragraph 2.1 has been characterized in the microwave range in terms of the scattering matrix by using a Vector Network Analyzer, VNA (Anritsu 37347C, Atsugi, Japan). The critical point in the measurement set-up is the transition between the microstrip under test and the coaxial cables connected to the two ports of the VNA. In practical cases, in spite of a careful design of the microstrip-cable transition, mismatch between the coaxial cables and the microstrip causes non-negligible errors in both reflections and transmission measurements, whose reduction requires proper error correction techniques. These techniques model the microwave circuit connected to the microstrip (including the transitions) as a linear network, allowing us to correct the systematic errors affecting the measurements [35,36]. This method, known as ‘VNA calibration process’, requires the use of known, or partially known, loads. The effectiveness of the method is affected mainly by two factors. The first one is related to the accuracy of the load used in the calibration process, while the second one depends on the stability (i.e., invariance) of the microwave set-up during the calibration and the measurement of DUT.

**Figure 4:**
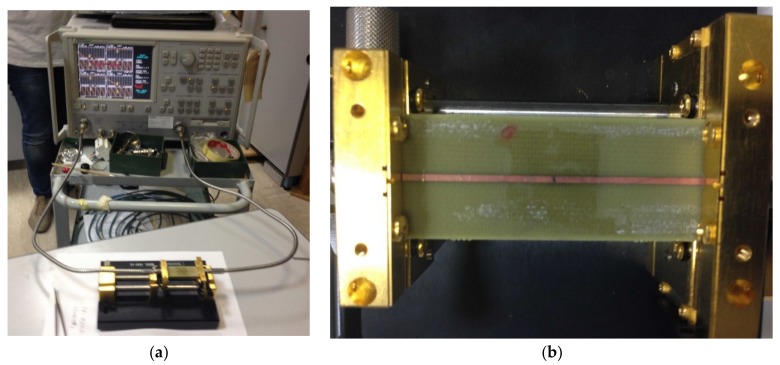
Setup for the microwave range characterization. (**a**) overall system; (**b**) details of the microstrip inserted into the text-fixture.

The critical point is the repeatability of the microstrip-coaxial cable transition mismatch when the different loads and the DUTs are measured. In the measurement campaign a high precision microstrip text fixture (Anritsu Universal Test Fixture 3680-20, Atsugi, Japan) has been adopted, while the calibration has been performed by using high-precision microstrip loads (available in the Anritsu 36804B-25M calibration kit, Atsugi, Japan). The text fixture has been connected to the VNA by means of semi-rigid phase stable cables. The VNA has been calibrated using Transmission-Reflection-Load (TRL) technique. In particular, 10 mm and 12 mm lines, as well as short circuits on both ports have been employed for the calibration, in order to achieve a very stable measurement up to 15 GHz. The measurement set-up is shown in Figure 4.

After the calibration, its accuracy has been verified by means of the measurement of a known load (a matched circuit on both ports); for each DUT the measurement has been taken two times, rotating the sample in the fixture in order to check for sample asymmetries. The calibration-verification-measurements procedure has been repeated at least three times for every sample batch. 

## 3. Results and Discussion

### 3.1. Structural Characterization of the Nanomaterial and of the Circuit

**Figure 5:**
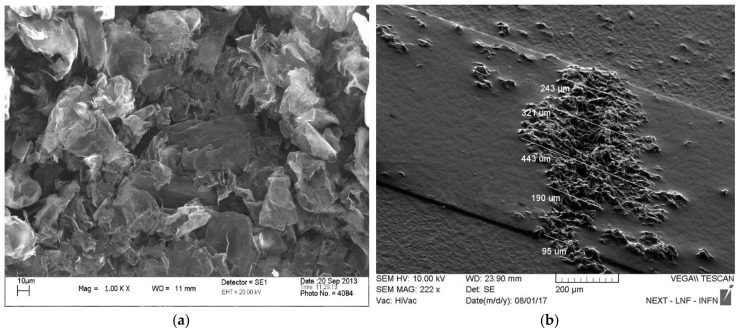
SEM images of: (**a**) GNP powder at a magnification 1000×; (**b**) GNP contact created after the self-assembly process (sample 2, with estimation of the extra length).

After the SEM analysis, the thickness of the GNPs were found to be smaller than 100 nm: Figure 5a shows an image of these flakes in powder. 

In addition, the mean composition identified by the SEM/EDX analysis highlights a non-negligible presence of oxygen and of impurities, such as Ca, S, Si, Al and other elements (see Table 2), which is the typical case for a commercial low-cost graphene material.

Figure 5b shows the results obtained after creating the GNP contact with the self-assembly procedure described in Section 2, highlighting the presence of GNPs exceeding the gap. An estimation of the lengths and heights of the box of the extra material to be considered in the model (see Figure 3) has been made by using SEM images, and the resulting ranges are reported in Table 3. 

### 3.2. Electromagnetic Simulations and Sensitivity Analysis

**Figure 6:**
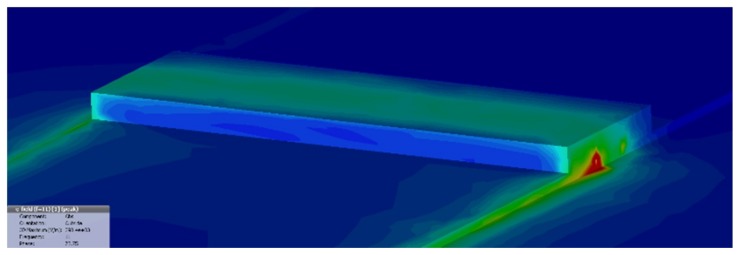
Computed distribution of the magnitude of the electric field at a frequency of 11 GHz.

The electromagnetic model presented in Section 2 has been used to simulate the system in the microwave frequency range, to retrieve the scattering parameters: Figure 6 shows the distribution of the electric field at 11 GHz, putting on evidence the non-negligible influence of the extra material. 

**Figure 7:**
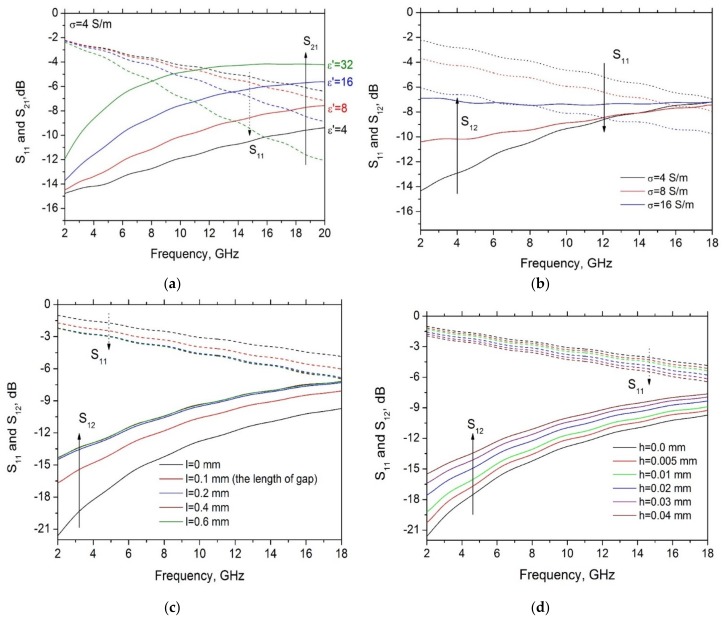
Computed scattering parameters *S*_11_ and *S*_12_ in the microwave range when varying: (**a**) the dielectric constant and (**b**) the conductivity of the GNP contact; (**c**) the length and (**d**) the height of the box of extra-material deposited on the gap.

A sensitivity analysis has then been carried out to investigate the dependence of the scattering parameters on each of the four degrees of freedom, and the results are plotted in Figure 7. Here, the following set of nominal values have been assumed: $\varepsilon^{\prime }$ = 10, σ=4 S/m, *l* = 0.4 mm and *h* = 0.06 mm. In each of the four plots all but one of these values are kept fixed. 

The reflection parameter *S*_11_ is: always decreasing with the frequency; decreasing with $\varepsilon^{\prime }$ at high frequencies but mostly insensitive to it at low frequencies (Figure 7a); decreasing with the conductivity σ by a factor almost independent on frequency (Figure 7b); decreasing with the length *l* by a factor almost independent on frequency up to values of about 2x the gap length, being insensitive to further length increase (Figure 7c); decreasing with the height *h* by a factor almost independent on frequency (Figure 7d).

The transmission parameter *S*_12_ is always increasing with the frequency; increasing with $\varepsilon^{\prime }$ (Figure 7a); increasing with the conductivity σ at low frequencies but mostly insensitive to it at high frequencies (Figure 7b); increasing with the length *l* by a factor almost independent on frequency up to values of about 2× the gap length, being insensitive to further length increase (Figure 7c); increasing with the height *h* by a factor almost independent on frequency (Figure 7d). 

### 3.3. Electromagnetic Characterization and Results Discussion

**Figure 8:**
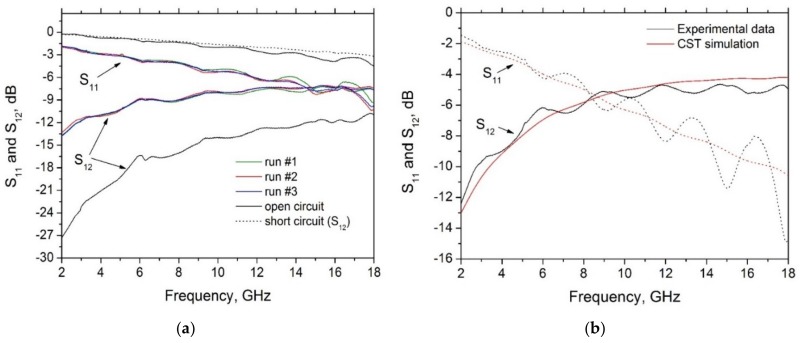
Experimental characterization of the test-vehicles in the microwave range: (**a**) measured S-parameters for Sample #1, for three different runs; (**b**) measured vs simulated S-parameters for Sample #2.

The result of the characterization in the microwave range is reported in Figure 8. Specifically, Figure 8a refers to Sample #1 and to the reference circuits used for the calibration (short and open circuit). The measurements have been repeated three times with no significant difference, hence assessing a good level of reproducibility (see Figure 8a).

The final step is the identification of the electrical parameters by comparing the simulation results to the experimental ones. To this end, recalling that the model is based on four degrees of freedom, $\varepsilon^{\prime }$, σ, *l* and *h*, a preliminary analysis is carried out to reduce the range of the values of at least three of these parameters. The geometrical parameters are bounded by the values provided in Table 3. As for the conductivity, a preliminary estimation of its DC value has been performed by measuring the DC resistance with a four-probe technique, providing the values in Table 4. In the following we assume that the conductivity in the microwave range remains of the same order of magnitude of the DC value.

Therefore, the identification procedure can be now carried out by varying the real part of the permittivity (the dielectric constant) $\varepsilon^{\prime }$ in order to match the simulations with the measurements, imposing that the geometrical parameters *l* and *h* fall in the ranges in Table 3, and that the conductivity σ remains in the order of magnitude reported in Table 4. An example of this procedure is reported in Figure 8b, referring to the sample #2. After analyzing the two samples, the estimated values of the dielectric constant $\varepsilon^{\prime }$ and of the conductivity σ in the considered range are finally reported in Table 4.

As expected, the low-cost fabrication and integration procedure cannot provide a strict control over the final electrical parameters, hence the permittivity values for the two test-vehicles are significantly different, although they remain of the same order of magnitude. 

The electrical conductivity value for the GNP contact in the arrangement presented in this paper is much lower than that measured for instance in isolated GNP films, with typical values of the order of $\sigma \sim 10^{4}$ S/m [12], that can rise even up to $\sigma \sim 10^{5}$ S/m [25]. This result is due to two main reasons: the first and probably the most relevant is the presence of the contact resistance at the GNP/Cu interfaces, which strongly reduces the overall conductivity, as is well known from the studies on carbon interconnects [28]. The second reason, as pointed out in [12], may be found in the degradation phenomena associated to the granularity, impurities and tunneling transport. The very low value of the equivalent electrical conductivity makes this solution not suitable for fabricating high-frequency interconnects. 

The results in Table 4 demonstrate that the GNP contact in the arrangement presented here exhibits a value of the real part of the permittivity that is higher than the values for isolated graphene sheets, typically below 10. From this point of view, this material behaves like the GNP-nanocomposites, that have been proven to exhibit huge permittivity values [30,37]. As a conclusion, this kind of material can be proposed when high values of permittivity are required, such as in nano-antennas or in off-chip transitions supporting quasi-Tranverse Electromagnetic Modes.

## 4. Conclusions

This paper has investigated the high-frequency equivalent electrical parameters of a contact realized by embedding a low-cost version of graphene, i.e., graphene nanoplatelets (GNPs) into a microstrip-like circuit. The GNPs have been embedded by means of an industrially appealing self-assembly procedure. The above assumptions introduce two main issues: the effect of the contacts between the GNPs and the copper electrodes and the limited control on the final shape of the contact.

In the microwave frequency range (2–18 GHz) the estimated equivalent conductivity ranges from 4 to 10 S/m, that is 3–4 orders of magnitude lower than the typical values expected for isolated GNP films. The equivalent relative dielectric constant falls in the range 23–40, which is comparable to the values obtained, for instance, in GNP nanocomposites.

The above results allow us to outline some perspectives about the potential applications of GNP contacts for high-frequency circuits. Indeed, there are at least two possible applications where the proposed circuit devices may be exploited: the use of embedded GNPs into a microstrip circuit as an attenuator element (demonstrated in [26]) or into the electrical package of an integrated circuit (IC), as a novel interposer material. The interposer layers are used in the so-called 3D integration technology to fan-out the IC pins with redistribution layers and packages circuits [38]. To improve the performance in terms of signal integrity, the interposer should exhibit a low electrical conductivity and a high dielectric constant, which is exactly the case of the contact analyzed in this paper.

## Figures and Tables

**Table 1:** Values of the geometrical parameters for the microstrip in Figure 2.

*A* (mm)	*B* (mm)	*W* (mm)	*H* (mm)	*L* (mm)
50	20	1.0	0.5	0.1

**Table 2:** Composition of the graphene nanoplatelets powder detected by SEM/EDX.

**Element**	C	O	Fe	Na	Mg	Al	Si	S	Ca	Cr
**Content (%)**	87.10	10.90	0.13	0.16	0.13	0.24	0.59	0.38	0.21	0.11

**Table 3:** Measured ranges of the values of the extra-material box.

Dimension	Sample 1	Sample 2
h (µm)	10–20	48–55
l (mm)	0.10–0.44	0.10–0.50

**Table 4:** Measured (in DC) and estimated values of the electrical parameters.

Parameter	Sample 1	Sample 2
*σ*(_DC_) (S/m)	2.4	2.5
*σ* (S/m)	4	10
$\varepsilon^{\prime }$	23	40

## References

[1] International Technology Roadmap for Semiconductors, ITRS. www.itrs2.net.

[2] Saito R., Dresselhaus G., Dresselhaus M.S. (2004). Physical Properties of Carbon Nanotubes.

[3] Castro Neto A.H., Guinea F., Peres N.M.R., Novoselov K.S., Geim A.K. (2009). The electronic properties of graphene. Rev. Mod. Phys..

[4] Bellucci S. (2005). Carbon nanotubes: Physics and applications. Phys. Status Solidi A.

[5] Avouris P., Chen Z., Perebeinos V. (2007). Carbon Based Electronics. Nat. Nanotechnol..

[6] Torrisi F., Hasan T., Wu W., Sun Z., Lombardo A., Kulmala T.S., Hsieh G.W., Jung S., Bonaccorso F., Paul P.J. (2012). Inkjet-printed Graphene Electronics. ACS Nano.

[7] Morris J.E., Iniewski K. (2013). Graphene, Carbon Nanotubes, and Nanostructures: Techniques and Applications.

[8] Jariwala D., Sangwan V.K., Lauhon L.J., Marksab T.J., Hersam M.C. (2013). Carbon nanomaterials for electronics, optoelectronics, photovoltaics, and sensing. Chem. Soc. Rev..

[9] Kuzhir P., Paddubskaya A., Bychanok D., Nemilentsau A., Shuba M., Plusch A., Maksimenko S., Bellucci S., Coderoni L., Micciulla F. (2011). Microwave probing of nanocarbon based epoxy resin composite films: Toward electromagnetic shielding. Thin Solid Films.

[10] Bychanok D.S., Plyushch A.O., Gorokhov G.V., Bychanok U.S., Kuzhira P.P., Maksimenko S.A. (2016). Microwave Radiation Absorbers Based on Corrugated Composites with Carbon Fibers. Techn. Phys..

[11] Plyushch A., Macutkevic J., Kuzhir P., Banys J., Bychanok D., Lambin P., Bistarelli S., Cataldo A., Micciulla F., Bellucci S. (2016). Electromagnetic properties of graphene nanoplatelets/epoxy composites. Compos. Sci. Technol..

[12] Sarto M.S., D’Aloia A.G., Tamburrano A., De Bellis G. (2017). Synthesis, Modeling, and Experimental Characterization of Graphite Nanoplatelet-Based Composites for EMC Applications. IEEE Trans. Electromagn. Compat..

[13] Li H., Xu C., Srivastava N., Banerjee K. (2009). Carbon Nanomaterials for Next-Generation Interconnects and Passives: Physics, Status, and Prospects. IEEE Trans. Electron Dev..

[14] Todri-Sanial A., Dijon J., Maffucci A. (2016). Carbon Nanotubes for Interconnects: Process, Design and Applications.

[15] Slepyan G.Y., Boag A., Mordachev V., Sinkevich E., Maksimenko S., Kuzhir P., Miano G., Portnoi M.E., Maffucci A. (2015). Nanoscale Electromagnetic Compatibility: Quantum Coupling and Matching in Nanocircuits. IEEE Trans. Electromagn. Compat..

[16] Maffucci A. (2009). Carbon nanotubes in nanopackaging applications. IEEE Nanotechnol. Mag..

[17] Morris J.E. (2018). Nanopackaging: Nanotechnologies and Electronics Packaging.

[18] Franklin A.D., Luisier M., Han S.-J., Tulevski G., Breslin C.M., Gignac L., Lundstrom M.S., Haensch W. (2012). Sub-10 nm Carbon Nanotube Transistor. Nano Lett..

[19] Valitova I., Amato M., Mahvash F., Cantele G., Maffucci A., Santato C., Martel R., Cicoira F. (2013). Carbon nanotube electrodes in organic transistors. Nanoscale.

[20] Shuba M.V., Slepyan G.Y., Maksimenko S.A., Thomsen C., Lakhtakia A. (2009). Theory of multiwall carbon nanotubes as waveguides and antennas in the infrared and the visible regimes. Phys. Rev. B.

[21] Berres J.A., Hanson G.W. (2011). Multiwall carbon nanotubes at RF-THz frequencies: Scattering, shielding, effective conductivity, and power dissipation. IEEE Trans. Antennas Prop..

[22] Hartmann R.R., Kono J., Portnoi M.E. (2014). Terahertz science and technology of carbon nanomaterials. Nanotechnology.

[23] Dabrowska A., Bellucci S., Cataldo A., Micciulla F., Huczk A. (2014). Nanocomposites of epoxy resin with graphene nanoplates and exfoliated graphite: Synthesis and electrical properties. Phys. Status Solidi B.

[24] Maffucci A., Micciulla F., Cataldo A., Miano G., Bellucci S. (2016). Bottom-up Realization and Electrical Characterization of a Graphene-Based Device. Nanotechnology.

[25] Wu H., Drzal L.T. (2012). Graphene nanoplatelet paper as a light-weight composite with excellent electrical and thermal conductivity and good gas barrier properties. Carbon.

[26] Pierantoni L., Mencarelli D., Bozzi M., Moro R., Moscato S., Perregrini L., Micciulla F., Cataldo A., Bellucci S. (2015). Broadband Microwave Attenuator Based on Few Layer Graphene Flakes. IEEE Trans. Microw. Theory Tech..

[27] Yasir M., Savi P., Bistarelli S., Cataldo A., Bozzi M., Perregrini L., Bellucci S. (2017). A Planar Antenna with Voltage-Controlled Frequency Tuning Based on Few-Layer Graphene. IEEE Antennas Wirel. Propag. Lett..

[28] Wilhite P., Vyas A.A., Tan J., Yamada T., Wang P., Park J., Yang C.Y. (2014). Metal nanocarbon contacts. Semicond. Sci. Technol..

[29] Chiariello A.G., Miano G., Maffucci A. Size and temperature effects on the resistance of copper and carbon nanotubes nano-interconnects. Proceedings of the IEEE 19th Conference on Electrical Performance of Electronic Packaging and Systems, EPEPS.

[30] Bellucci S., Bistarelli S., Cataldo A., Micciulla F., Kranauskaite I., Macutkevic J., Banys J., Volynets N., Paddubskaya A., Bychanok D. (2015). Broadband Dielectric Spectroscopy of Composites Filled with Various Carbon Materials. IEEE Trans. Microw. Theory Tech..

[31] Chen L.F., Ong C.K., Neo C.P., Varadan V.V., Varadan V.K. (2004). Microwave Electronics: Measurement and Materials Characterization.

[32] Anderson J.M., Sibbald C.L., Stuchly S.S. (1994). Dielectric Measurements Using a Rational Function Model. IEEE Trans. Microw. Theory Tech..

[33] CST Microwave Studio. https://www.cst.com.

[34] Griffiths D.J. (1999). Introduction to Electrodynamics.

[35] Bryant G.H. (1988). Principles of Microwave Measurements.

[36] Silvonen K.J. (1992). A General Approach to Network Analyzer Calibration. IEEE Trans. Microw. Theory Tech..

[37] Li Y.C., Tjong S.C., Li R.K.Y. (2010). Electrical conductivity and dielectric response of poly(vinylidene fluoride)–graphite nanoplatelet composites. Synth. Met..

[38] Swaminathan M., Han K.J. (2014). Design and Modeling for 3D ICs and Interposers.

